# OPTIMISM AND EXPECTATIONS REGARDING AGING AMONG YOUNG ADULTS AND OLDER ADULTS

**DOI:** 10.1093/geroni/igz038.1711

**Published:** 2019-11-08

**Authors:** Samuel C Van Vleet, Jenna M Moore, Sarah Edzards, Leah N Smith, Jennifer Sawyer, Allyson M Coldiron, Allie M Sandlin, Michael D Barnett

**Affiliations:** 1 University of North Texas, Denton, United States; 2 The University of Texas at Tyler, Tyler, Texas, United States

## Abstract

A large body of research has found that individuals’ attitudes toward aging may influence their future health. Previous research has found that age is associated with more negative expectations toward aging. However, it is possible that optimism, or generalized positive expectancies regarding future outcomes may play a role in expectations regarding aging. Optimism has been identified as a key component of successful aging. The purpose of this study was to compare expectations regarding aging among young adult and older adult age cohorts, controlling for optimism, and to investigate the differential relationships between optimism and expectations regarding aging by age cohort. Young adults (n = 130) and older adults (n = 335) completed a survey containing the Expectations Regarding Aging – 12 and the Optimism-Pessimism Scale. Results found that, after controlling for optimism, older adults endorsed more negative expectations regarding aging. Comparison of the correlation coefficients between optimism and expectations regarding aging among age cohorts found that optimism was significantly associated with expectations regarding aging among older adults but not young adults and that this difference was significant. Taken together, the results suggest that older adults have more negative expectations for aging and that optimism may play a key role in older adults’ expectations regarding aging.

